# Supplementary material for: Extracellular Vesicles from Bothrops jararaca Venom Are Diverse in Structure and Protein Composition and Interact with Mammalian Cells
Source: Toxins (Basel). 2022 Nov 19;14(11):806. doi: 10.3390/toxins14110806 (PMC9698812; doi:10.3390/toxins14110806)
Supplement: Supplementary file 1 [file toxins-14-00806-s001.zip › Supplementary Material Figures S1-S11 and Table S1 (1).pdf]

Supplementary Material: Extracellular vesicles from *Bothrops jararaca venom* are diverse in structure, protein composition and interact with mammalian cells

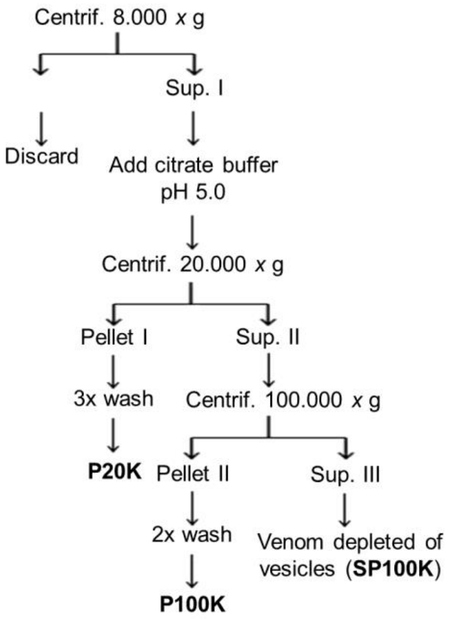

Figure S1. Workflow of extracellular vesicles isolation. The cellular debris was removed by centrifugation at 8,000 x g for 25 min. Fraction P20K was obtained after centrifugation at 20,000 x g for 25 min. After that, fraction P100K was obtained after centrifugation at 100,000 x g for 2 h. Venom depleted of vesicles was defined as the P100K supernatant (SP100K).

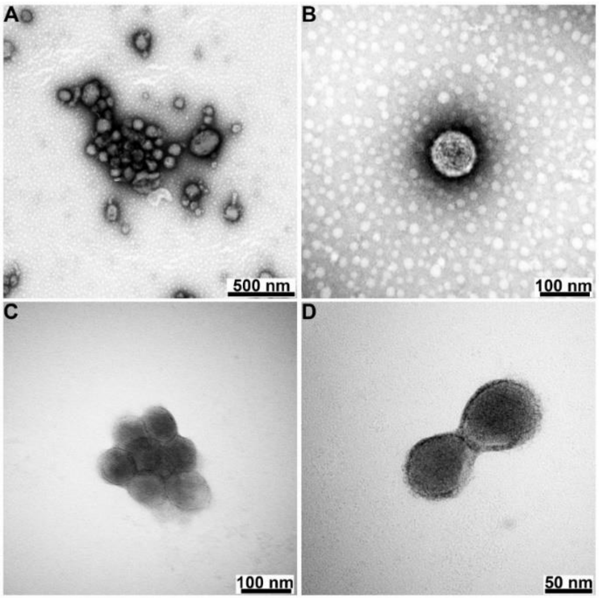

Figure S2. Negative Staining analysis of Bj-EVs. Representative images of integral Bj-EVs (P20K - A,B) and (P100K- C,D) contrasted and visualized by transmission electron microscopy (TEM). Images were used to measure EV size using ImageJ. Relative to main figure 1-I.

Spot ID	MW (kDa)	m/z	z	Peptide sequence	Score	Best NCBI match	Protein family
**P20K**	70	604	3	GSFELTILHTNDVHAR	857	*Protobothrops elegans* BAP39925	5'-nucleotidase
		868.4	3	YDAMoxALGNHEFDNGLAGLLDPLLK			
		526.6	3	HANFPILSANIRPK			
		1042.5	3	ETPVLSNPGPYLEFRDEVEELQNHANK			
		778.4	2	IIALGHSGFLEDQR			
		653.8	2	QVPVVQAYAFGK			
		1211.1	2	FHECNLGNLICDAVIYNNVR			
		785.4	2	HGQGMGELLQVSGIK			
		725.4	2	VVSLNVLCTECR			
		1330.7	2	GYDAMoxALGNHEFDNGLAGLLDPLLK	739 (52)	*Gloydius brevicaudus* B6EWW8	5'-nucleotidase
		654.7	3	LLLPSFLAGGGDGYHMoxLKG			
		450.2	3	VVYDLSQKPGSR	204 (50)	*Macrovipera lebetina* AHJ80886	5'-nucleotidase
		596.6	3	IINEPTAAAIAYGLDKK	93	*Orcinus orca* XP_012391632	heat shock cognate 71 kDa
		769.4	2	RAGFEELNADLFR			
**P100K**	70	672.3	2	CTGQDCYGGVAR	479	*Gloydius brevicaudus* B6EWW8	5'-nucleotidase
		526.6	3	HANFPILSANIRPK			
		476.3	2	VGIIGYTTK			
		859.9	2	ETPVLSNPGPYLEFR			
		653.9	2	QVPVVQAYAFGK			
		578.8	2	SSGNPILLNKN			
		785.4	2	HGQGMoxGELLQVSGIK			
		725.3	2	VVSLNVLCTECR			
		635.7	3	LLLPSFLAGGGDGYHMoxLK			
		450.2	3	VVYDLSQKPGSR	288 (66)	*Macrovipera lebetina* AHJ80886	5'-nucleotidase
		744.4	2	TTPSYVAFTDTER	113	*Oxyuranus s. scutellatus* AAY33973	HSP70
		615.3	2	DAGTITGLNVLR			

Figure S3: *In-gel* protein digestion and mass spectrometry. Digested protein gel bands (related to main Figure 2) were analyzed in a Q-TOF mass spectrometer and deconvoluted exported data were identified in the open Mascot MS/MS Ions Search. The search parameters were set as follows: up to 2 missed cleavages; enzyme semi-trypsin; taxonomy - all entries; fixed modification - carbamidomethyl in cysteines; variable modifications - oxidation of methionine; peptide tol: ± 0.1 Da; MS/MS tol: 0.2 Da. Scores are relative to full protein identification while the score in parentheses refers to individual non-redundant ion. Only identifications with at least 2 peptides or a score over 100 were considered. m/z = mass to charge ratio; z = charge of the ion; Mox = oxidized methionine. HSP70 = heat shock protein 70

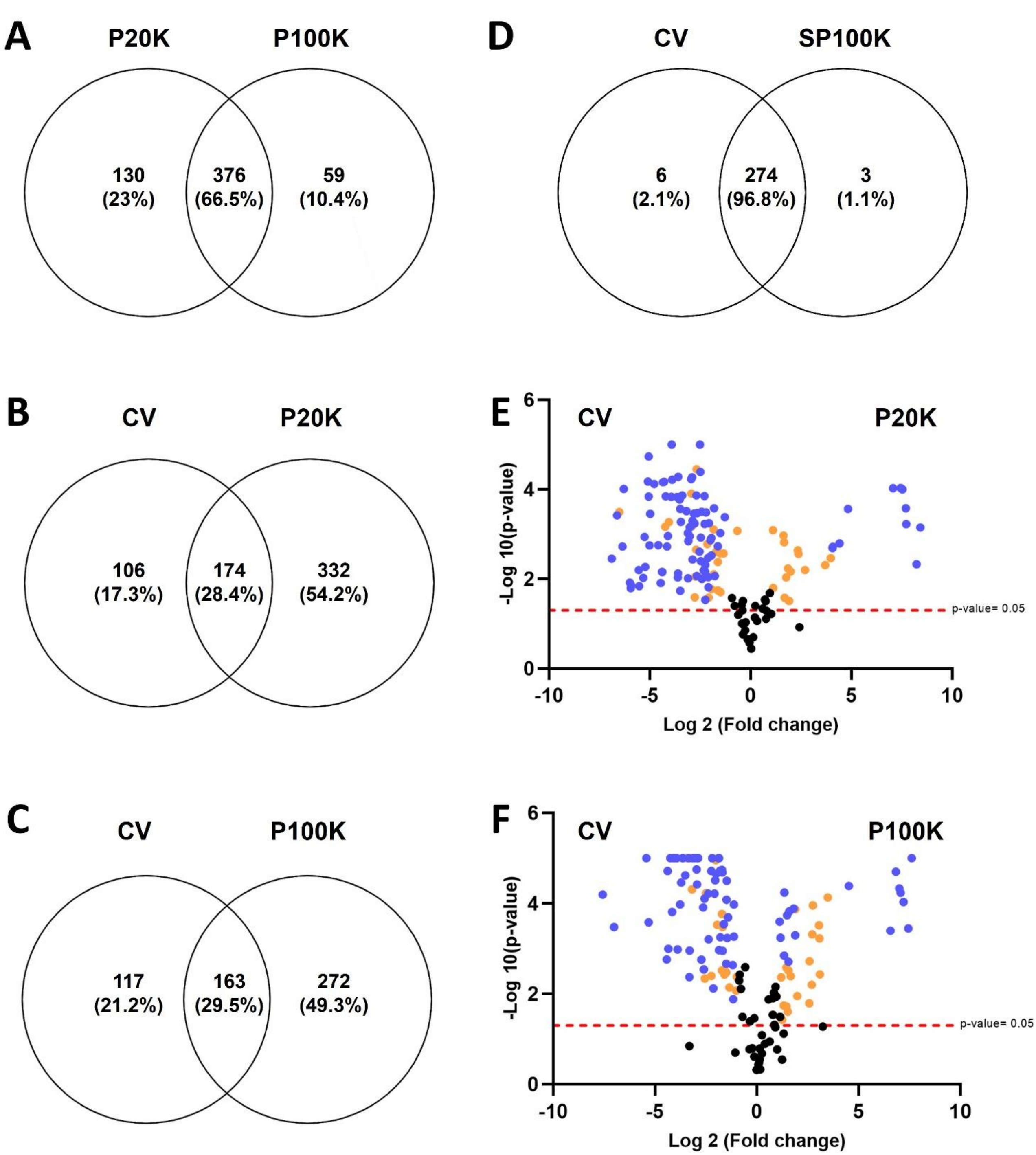

Figure S4. Shotgun proteomics of venom fractions. Venn Diagrams (A-D) showing common protein identified among venom fractions. Volcano Plots (E,F) show quantitative differences. Blue dots represent significant quantitative differences. Orange dots were originally blue but filtered out by the L-stringency in the T-Fold algorithm (related to low abundance signals of proteins). CV = crude venom; P20K and P100K = vesicles pelleted at 20,000 x g and 100,000 x g, respectively; SP100K = supernatant of P100K or venom depleted of vesicles.

Table S1: EV-markers identified in Bj-EVs. Upper case numbers indicate the references for the EV markers. Most of the markers are also listed in the Vesiclepedia [1] and EV-track [2] databases.

Biological Roles	EV-markers	Positive Samples
EV Biogenesis	ALIX [3-7]	P20K, P100K
	Syntenin [3-8]	P20K, P100K
	CHMP5 [7,9,10]	P100K
	Clathrin [1,7]	P100K
Signal transduction	14-3-3 [6,7]	P20K, P100K
	G proteins [6,7]	P20K, P100K
	Protein kinases [1,7]	P20K, P100K
Cytoskeletal	Actin [6-8,11]	P20K, P100K
	Cofilin [6,12]	P20K, P100K
	Profilin [1,12]	P20K, P100K
Membrane organization and trafficking	Flotillin [6-8,11]	P20K, P100K
	Annexin A1 [10,13]	P20K
	RABs [6,7]	P20K, P100K
MVE fusion with PM	SNAP23 [7,8,12]	P20K, P100K
	Syntaxin-3 [7,14,15]	P20K
	Synaptotagmin-like proteins [7,16]	P20K, P100K
RNA-binding proteins	Y-box-binding protein 1 [7,8,17,18]	P100K
	Polyadenylate-binding protein [8,19]	P100K
	Regulator of nonsense transcripts 1 [8]	P100K
	Heterogeneous nuclear ribonucleoprotein Q [8,18]	P20K, P100K
	ELAV-like protein 1 [8,18]	P100K
	GTP-binding nuclear protein Ran-like [8,19]	P20K
Others	Chloride intracellular channel protein 1 [1,12]	P20K, P100K
	Heat shock proteins [6-8,11,12]	P20K, P100K
	DnaJ homolog subfamily C [20,21]	P20K, P100K

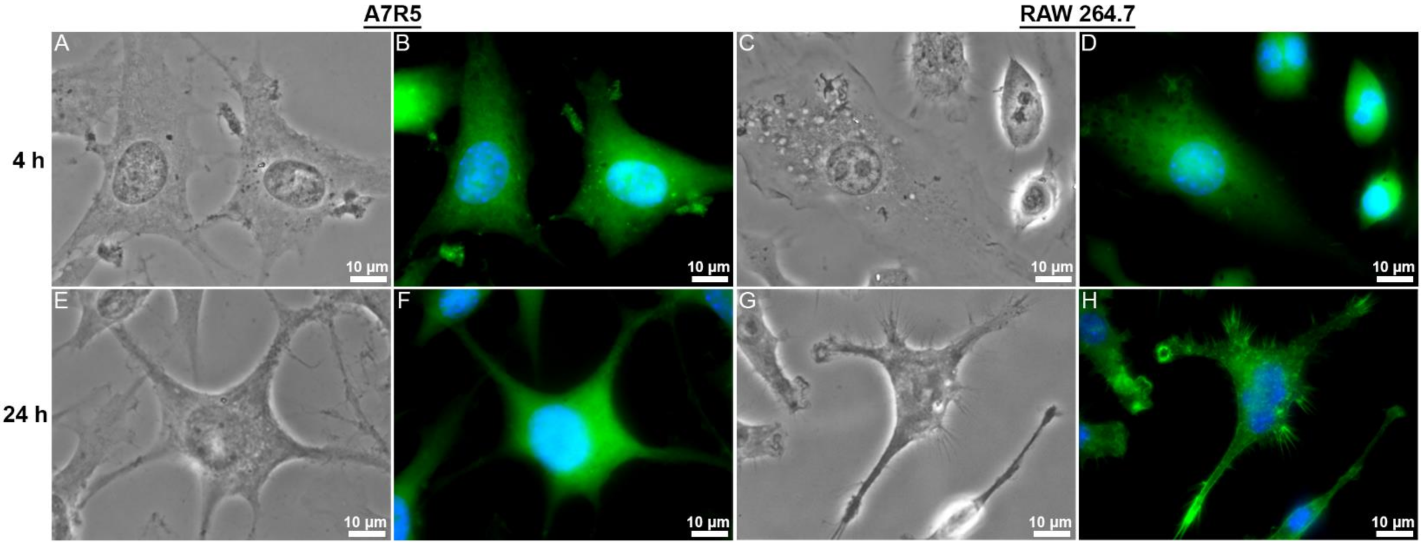

Figure S5- Fluorescent optical microscopy of A7R5 muscle and RAW 264.7 control cells. The images are control cells related to main Figures 4 and 5. All cells were maintained in the same conditions, except by the Bj-EV-treatment. Images of differential interference contrast (DIC) (A,C,E,G) and the maximum projection of images from different focal planes after the 3D deconvolution process (B,D,F,H) are presented. A7R5 4h (A,B) and 24 h (E,F) cell culture; RAW 264.7 4h (C,D) and 24h (G,H) cell culture. Green = actin (phalloidin); Blue = nuclei (Hoechst).

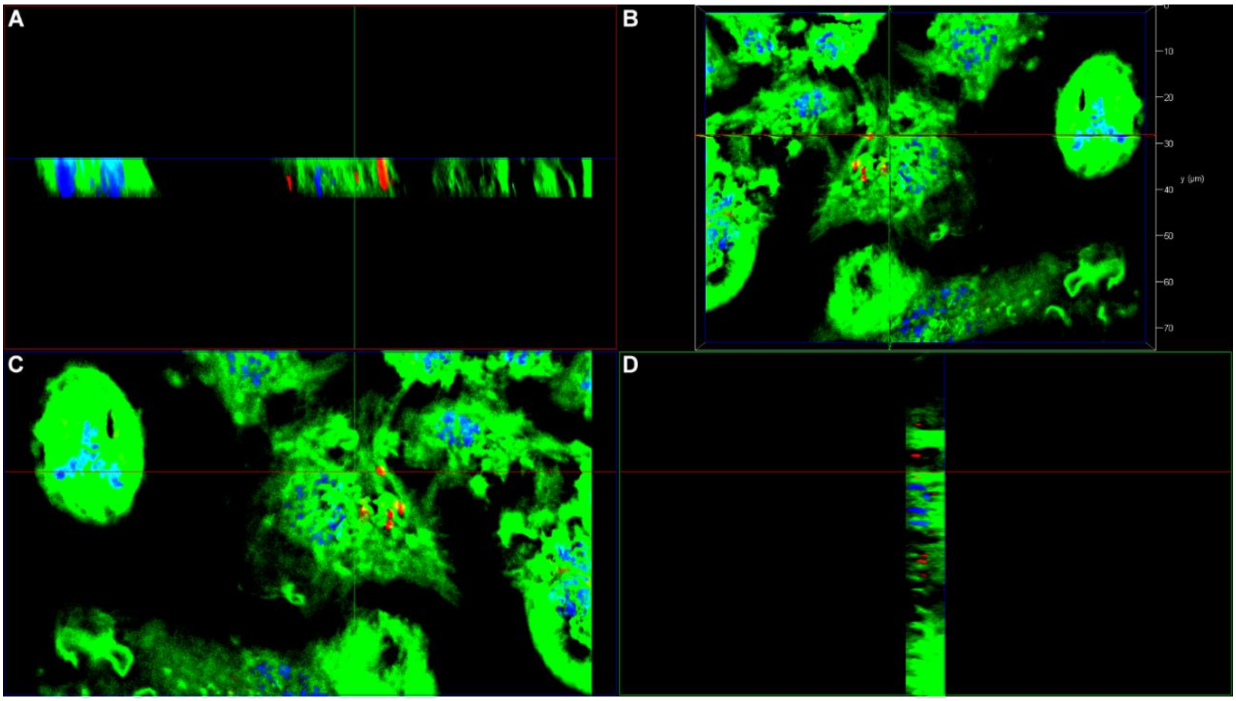

Figure S6. Axis projections of RAW 264.7 macrophages 24h treatment with P20K. Three-dimensional reconstruction obtained from 3D deconvolution using LAS X software v. 3.2.1.9702 after scanning different focal planes. Bj-EVs (red) can be observed together with cellular cytoskeleton (green), sometimes next to the nuclei (blue), suggesting internalization. Images were obtained using a Leica DMI 6000B fluorescence microscope. Red = P20K (Dil); Green = actin (phalloidin); Blue = nuclei (Hoechst).

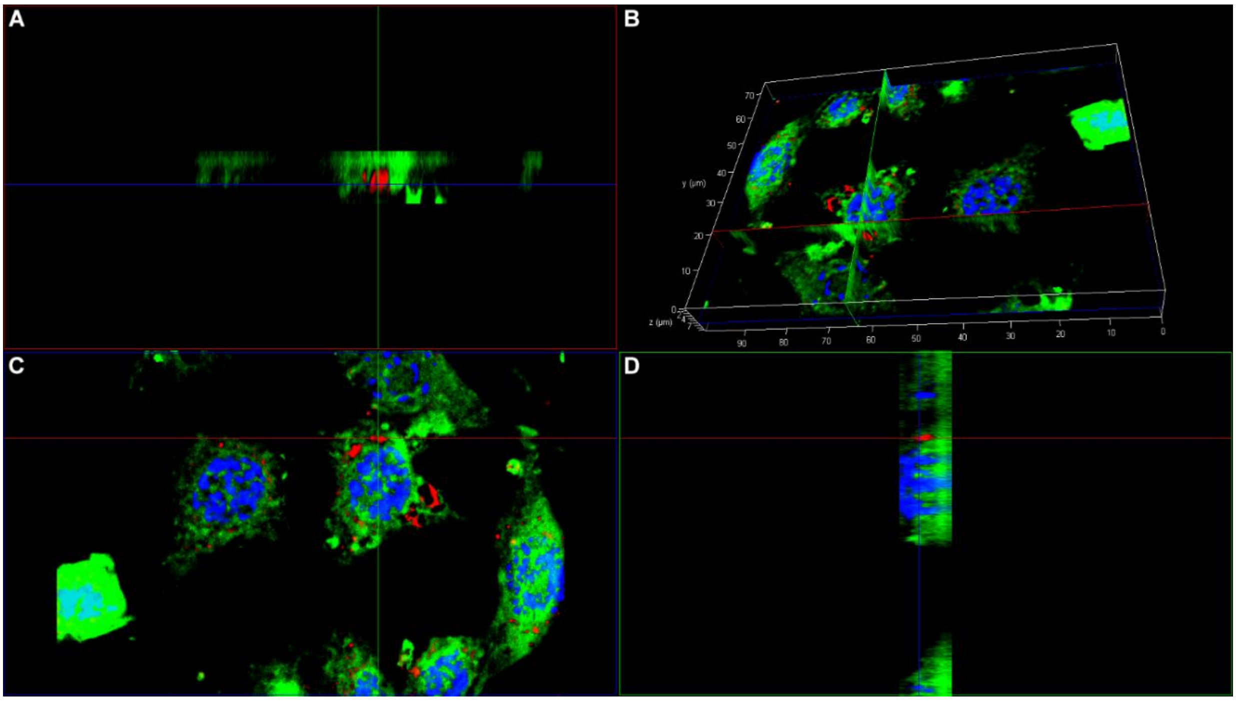

Figure S7. Axis projections of A7R5 muscle cells after 24 h treatment with P20K. Three-dimensional reconstruction obtained from 3D deconvolution using LAS X software v. 3.2.1.9702 after scanning different focal planes. Bj-EVs (red) can be observed together with cellular cytoskeleton (green), sometimes next to the nuclei (blue), suggesting internalization. Images were obtained in a Leica DMI 6000B fluorescence microscope. Red = P20K (Dil); Green = actin (phalloidin); Blue = nuclei (Hoechst).

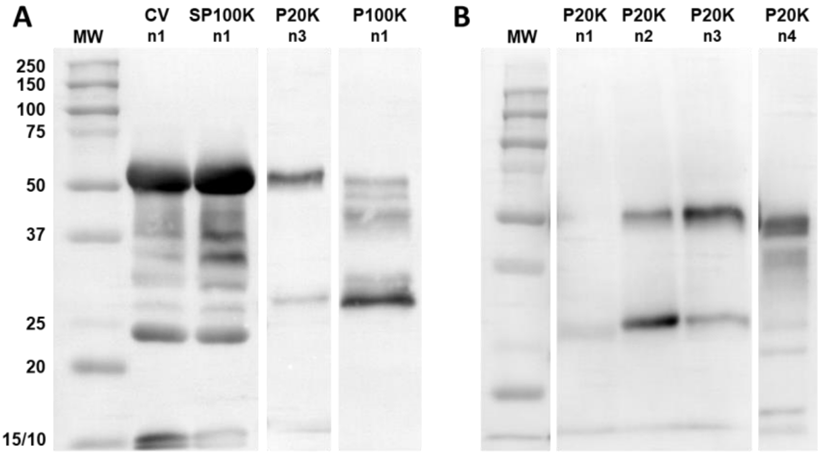

Figure S8: Western Blot of *B. jararaca* venom fractions collected from different animal pools. Each lane corresponds to a different venom extraction, from distinct animal groups (n1 to n4). In the main manuscript, the SDS-PAGE Figure 2 refers to n1. (A) Western Blot of *B. jararaca* venom and fractions (10 μg), using polyclonal antibothropic serum (ABS – Instituto Vital Brazil) as the first antibody (1:4000, 2h). The P20K lane refers to n3 because it was more representative. (B) Western Blot of *B. jararaca* P20K fractions (10 μg), using polyclonal antibothropic serum (ABS – Instituto Vital Brazil) as the first antibody (1:5000, 2h). MW = molecular weight; CV = crude venom; SP100K = supernatant of P100K, or venom depleted of vesicles; P20K = vesicles pelleted at 20,000xg; P100K = vesicles pelleted at 100,000xg.

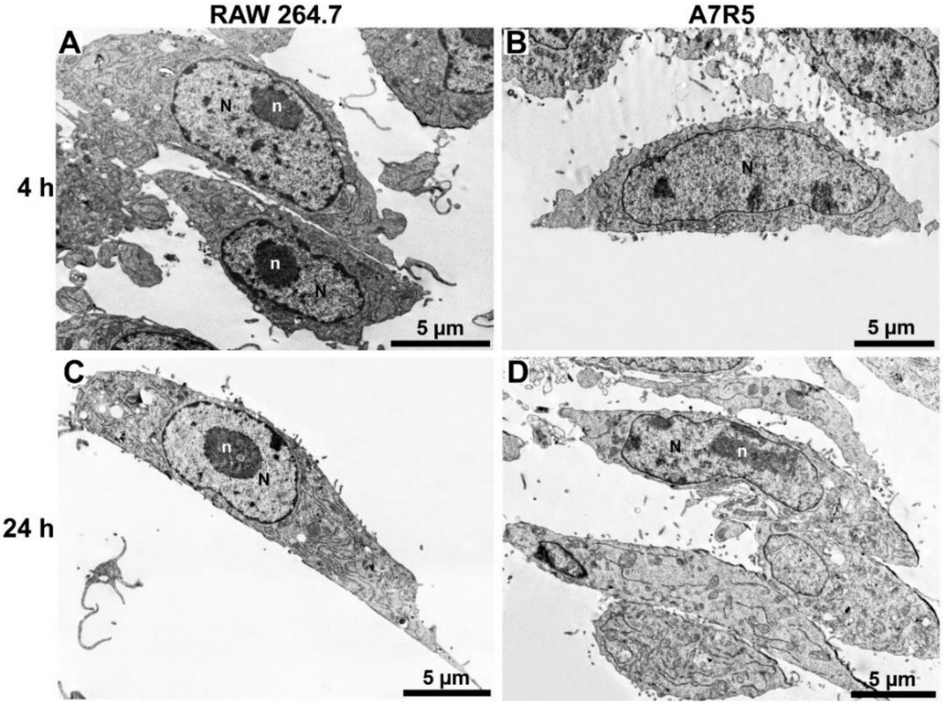

Figure S9: Ultrathin sections of macrophages and muscle cells without treatment with Bj-EVs (controls) obtained by <u>T</u>ransmission <u>E</u>lectron <u>M</u>icroscopy in <u>S</u>canning <u>E</u>lectron <u>M</u>icroscopy (STEM-IN-SEM). These images are the control macrophages (RAW 264.7) and muscle cells (A7R5) without treatment with Bj-EVs, as referred in Figure 7, Figure 8, Figure S10, and Figure S11. The control cells showed a regular ultrastructure. N = nucleus; n = nucleolus.

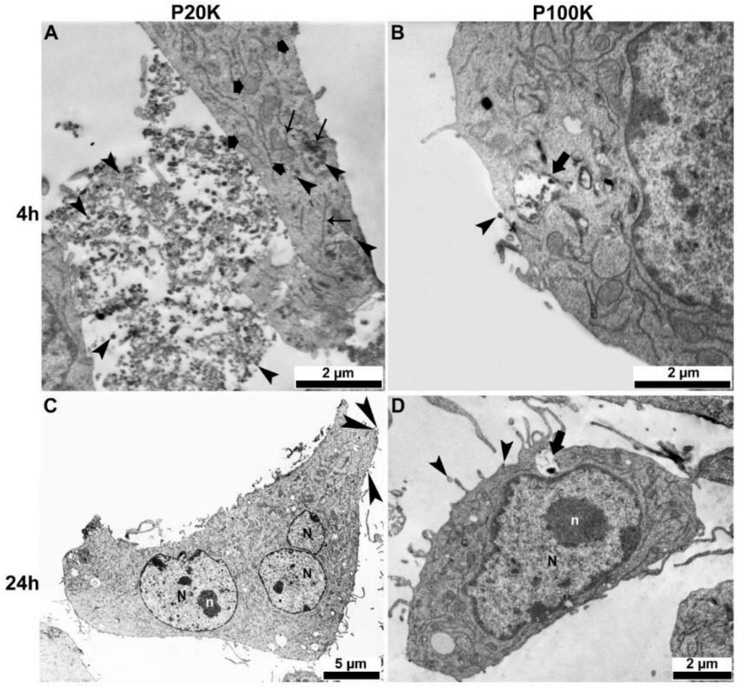

Figure S10: Ultrathin sections of RAW 264.7 macrophages treated with Bj-EVs. The macrophages were treated with Bj-EVs for 4 h (A,B) and 24 h (C,D). The arrowheads point to EVs interacting with the cell surface or inside the cytosol. Large vacuoles containing EVs (arrows) can be observed in panels B and D. The results suggest possible changes in the mitochondrion (Fig. A, small arrows) and the presence of an endoplasmic reticulum profile in close association with EVs (Fig. A, thin arrows). Images were obtained by Transmission Electron Microscopy in Scanning Electron Microscopy (STEM-IN-SEM). N = nucleus; n = nucleolus.

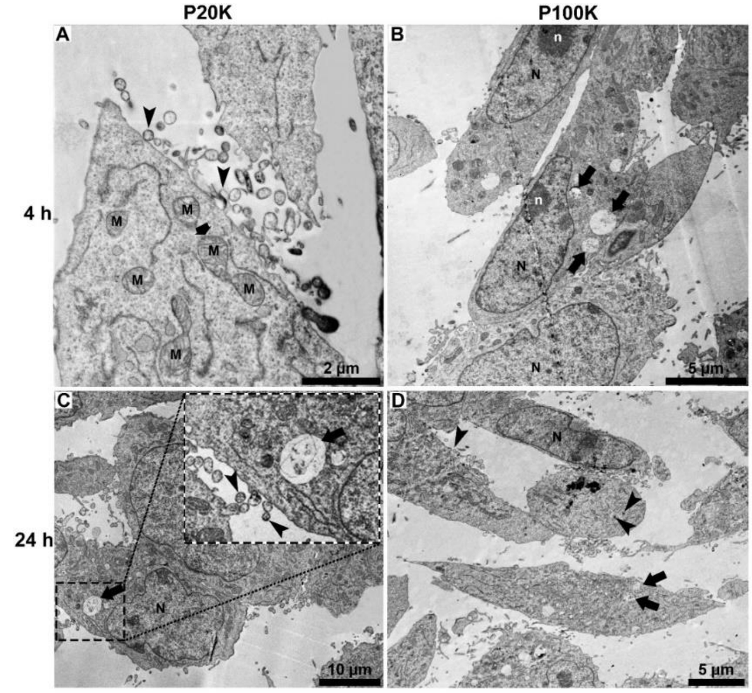

Figure S11: Ultrathin sections of A7R5 muscle cells treated with Bj-EVs. Muscle cells were treated with Bj-EVs for 4 h (A,B) and 24 h (C,D). Compared with the control cells, the images indicate possible alterations in organelles such as mitochondria (Panel A; small arrow) and the nucleus. Also, several EVs can be observed interacting with the cell surface or inside the cytosol (Panels A, C, D; arrowheads). Arrows indicate vacuoles loaded with vesicles (Panels B,D). Images were obtained by Transmission Electron Microscopy in Scanning Electron Microscopy (STEM-IN-SEM). N = nucleus; n = nucleolus; M = mitochondria.

References

1. Pathan, M.; Fonseka, P.; Chitti, S. V.; Kang, T.; Sanwlani, R.; Van Deun, J.; Hendrix, A.; Mathivanan, S. Vesiclepedia 2019: A compendium of RNA, proteins, lipids and metabolites in extracellular vesicles. Nucleic Acids Res. 2019, 47, D516–D519, doi:10.1093/nar/gky1029.

2. Van Deun, J.; Mestdagh, P.; Agostinis, P.; Akay, Ö.; Anand, S.; Anckaert, J.; Martinez, Z.A.; Baetens, T.; Beghein, E.; Bertier, L.; et al. EV-TRACK: Transparent reporting and centralizing knowledge in extracellular vesicle research. Nat. Methods 2017, 14, 228–232, doi:10.1038/nmeth.4185.

3. Baietti, M.F.; Zhang, Z.; Mortier, E.; Melchior, A.; Degeest, G.; Geeraerts, A.; Ivarsson, Y.; Depoortere, F.; Coomans, C.; Vermeiren, E.; et al. Syndecan-syntenin-ALIX regulates the biogenesis of exosomes. Nat. Cell Biol. 2012, 14, 677–685, doi:10.1038/ncb2502.

4. Ghossoub, R.; Lembo, F.; Rubio, A.; Gaillard, C.B.; Bouchet, J.; Vitale, N.; Slavík, J.; Machala, M.; Zimmermann, P. Syntenin-ALIX exosome biogenesis and budding into multivesicular bodies are controlled by ARF6 and PLD2. Nat. Commun. 2014, 5, doi:10.1038/ncomms4477.

5. Roucourt, B.; Meeussen, S.; Bao, J.; Zimmermann, P.; David, G. Heparanase activates the syndecan-syntenin-ALIX exosome pathway. Cell Res. 2015, 25, 412–428, doi:10.1038/cr.2015.29.

6. Colombo, M.; Raposo, G.; Théry, C. Biogenesis, Secretion, and Intercellular Interactions of Exosomes and Other Extracellular Vesicles. Annu. Rev. Cell Dev. Biol. 2014, 30, 255–289, doi:10.1146/annurev-cellbio-101512-122326.

7. Van Niel, G.; D'Angelo, G.; Raposo, G. Shedding light on the cell biology of extracellular vesicles. Nat. Rev. Mol. Cell Biol. 2018, 19, 213–228, doi:10.1038/nrm.2017.125.

8. Kugeratski, F.G.; Hodge, K.; Lilla, S.; McAndrews, K.M.; Zhou, X.; Hwang, R.F.; Zanivan, S.; Kalluri, R. Quantitative proteomics identifies the core proteome of exosomes with syntenin-1 as the highest abundant protein and a putative universal biomarker; Springer US, 2021; Vol. 23; ISBN 0000021547.

9. Gao, C.; Zhuang, X.; Shen, J.; Jiang, L. Plant ESCRT Complexes: Moving Beyond Endosomal Sorting. Trends Plant Sci. 2017, 22, 986–998, doi:10.1016/j.tplants.2017.08.003.

10. van Niel, G.; Carter, D.R.F.; Clayton, A.; Lambert, D.W.; Raposo, G.; Vader, P. Challenges and directions in studying cell–cell communication by extracellular vesicles. Nat. Rev. Mol. Cell Biol. 2022, 23, 369–382, doi:10.1038/s41580-022-00460-3.

11. Kowal, J.; Arras, G.; Colombo, M.; Jouve, M.; Morath, J.P.; Primdal-Bengtson, B.; Dingli, F.; Loew, D.; Tkach, M.; Théry, C. Proteomic comparison defines novel markers to characterize heterogeneous populations of extracellular vesicle subtypes. Proc. Natl. Acad. Sci. U. S. A. 2016, 113, E968–E977, doi:10.1073/pnas.1521230113.

12. Choi, D.; Go, G.; Kim, D.K.; Lee, J.; Park, S.M.; Di Vizio, D.; Gho, Y.S. Quantitative proteomic analysis of trypsin-treated extracellular vesicles to identify the real-vesicular proteins. J. Extracell. Vesicles 2020, 9, doi:10.1080/20013078.2020.1757209.

13. Jeppesen, D.K.; Fenix, A.M.; Franklin, J.L.; Higginbotham, J.N.; Zhang, Q.; Zimmerman, L.J.; Liebler, D.C.; Ping, J.; Liu, Q.; Evans, R.; et al. Reassessment of Exosome Composition. Cell 2019, 177, 428-445.e18, doi:10.1016/j.cell.2019.02.029.

14. Gradilla, A.C.; González, E.; Seijo, I.; Andrés, G.; Bischoff, M.; González-Mendez, L.; Sánchez, V.; Callejo, A.; Ibáñez, C.; Guerra, M.; et al. Exosomes as Hedgehog carriers in cytoneme-mediated transport and secretion. Nat. Commun. 2014, 5, doi:10.1038/ncomms6649.

15. Koles, K.; Nunnari, J.; Korkut, C.; Barria, R.; Brewer, C.; Li, Y.; Leszyk, J.; Zhang, B.; Budnik, V. Mechanism of evenness interrupted (Evi)-exosome release at synaptic boutons. J. Biol. Chem. 2012, 287, 16820–16834, doi:10.1074/jbc.M112.342667.

16. Ostrowski, M.; Carmo, N.B.; Krumeich, S.; Fanget, I.; Raposo, G.; Savina, A.; Moita, C.F.; Schauer, K.; Hume, A.N.; Freitas, R.P.; et al. Rab27a and Rab27b control different steps of the exosome secretion pathway. Nat. Cell Biol. 2010, 12, 19–30, doi:10.1038/ncb2000.

17. Shurtleff, M.J.; Temoche-Diaz, M.M.; Karfilis, K. V.; Ri, S.; Schekman, R. Y-box protein 1 is required to sort microRNAs into exosomes in cells and in a cell-free reaction. Elife 2016, 5, 1–23, doi:10.7554/eLife.19276.

18. Fabbiano, F.; Corsi, J.; Gurrieri, E.; Trevisan, C.; Notarangelo, M.; D'Agostino, V.G. RNA packaging into extracellular vesicles: An orchestra of RNA-binding proteins? J. Extracell. Vesicles 2020, 10, doi:10.1002/jev2.12043.

19. Sork, H.; Corso, G.; Krjutskov, K.; Johansson, H.J.; Nordin, J.Z.; Wiklander, O.P.B.; Lee, Y.X.F.; Westholm, J.O.; Lehtiö, J.; Wood, M.J.A.; et al. Heterogeneity and interplay of the extracellular vesicle small RNA transcriptome and proteome. Sci. Rep. 2018, 8, 1–12, doi:10.1038/s41598-018-28485-9.

20. Deng, J.; Koutras, C.; Donnelier, J.; Alshehri, M.; Fotouhi, M.; Girard, M.; Casha, S.; McPherson, P.S.; Robbins, S.M.; Braun, J.E.A. Neurons Export Extracellular Vesicles Enriched in Cysteine String Protein and Misfolded Protein Cargo. Sci. Rep. 2017, 7, 1–12, doi:10.1038/s41598-017-01115-6.

21. Rodríguez-Vega, A.; Losada-Barragán, M.; Berbert, L.R.; Mesquita-Rodrigues, C.; Bombaça, A.C.S.; Menna-Barreto, R.; Aquino, P.; Carvalho, P.C.; Padrón, G.; de Jesus, J.B.; et al. Quantitative analysis of proteins secreted by Leishmania (Viannia) braziliensis strains associated to distinct clinical manifestations of American Tegumentary Leishmaniasis. J. Proteomics 2021, 232, doi:10.1016/j.jprot.2020.104077.
